# Chinese Herbal Medicine on Cardiovascular Diseases and the Mechanisms of Action

**DOI:** 10.3389/fphar.2016.00469

**Published:** 2016-12-01

**Authors:** Cuiqing Liu, Yu Huang

**Affiliations:** ^1^Department of Preventive Medicine, Basic Medical College, Zhejiang Chinese Medical UniversityHangzhou, China; ^2^School of Biomedical Sciences, Institute of Vascular Medicine and Li Ka Shing Institute of Health Sciences, Chinese University of Hong KongHong Kong, China

**Keywords:** Chinese herbal medicine, hypertension, atherosclerosis, dyslipidemia, heart disease, cardiovascular diseases

## Abstract

Cardiovascular diseases are the principal cause of death worldwide. The potentially serious adverse effects of therapeutic drugs lead to growing awareness of the role of Chinese herbal medicine in the treatment of cardiovascular diseases. Chinese herbal medicine has been widely used in many countries especially in China from antiquity; however, the mechanisms by which herbal medicine acts in the prevention and treatment of cardiovascular diseases are far from clear. In this review, we briefly describe the characteristics of Chinese herbal medicine by comparing with western medicine. Then we summarize the formulae and herbs/natural products applied in the clinic and animal studies being sorted according to the specific cardiovascular diseases. Most importantly, we elaborate the existing investigations into mechanisms by which herbal compounds act at the cellular levels, including vascular smooth muscle cells, endothelial cells, cardiomyocytes and immune cells. Future research should focus on well-designed clinic trial, in-depth mechanic study, investigations on side effects of herbs and drug interactions. Studies on developing new agents with effectiveness and safety from traditional Chinese medicine is a promising way for prevention and treatment of patients with cardiovascular diseases.

Cardiovascular diseases, mainly including atherosclerosis, hypertension, cardiac hypertrophy, myocardial infarction and heart failure, are the principal cause of death worldwide. The increasing number of patients around the world suffering from CVDs indicates the need for innovative strategies for more effective prevention and treatment. Currently, western medicine, CHM and integrative medicine are the three major models of health care around the world. The western medicine has been considered as the mainstream medical treatment. For example, antithrombotic drugs combined with timely reperfusion therapy, coronary artery bypass grafting or precutaneous coronary intervention are widely applied to patients with CVDs. Although a widespread proof showed these therapeutic regimes effective in reducing cardiovascular events, the potentially serious adverse effects are still key challenges. Thus it prompted the search for alternative and complementary therapies for better management of CVDs. CHM generally applies some natural plant products including dietary factors or herbal medicines. There is growing awareness of the role of herbs in the prevention of CVDs and the possibility of their use in treatment (Li et al., 2015). With the more successful clinic application of CHM in CVDs prevention and treatment, the effects of CHM have drawn greater attention, even in developed countries including the United States and Australia. In this review, we focus on both the clinic practices and experimental studies to summarize the application of plant products and mechanistic investigations.

## Search Strategy

To review all studies directly describing the application of CHM in CVDs, we used the keywords “cardiovascular” and “herbs” with limiting the search to title and/or abstract. The initial search generated 281 results. We then afterward selected the articles based on our aims. Since there are lots of formulae widely used in clinic in China, Guidelines for Diagnosis and Treatment of Common Internal Diseases in Chinese Medicine was referred too. A total of 123 articles and 1 Chinese Guideline was finally included in the review.

## Comparison Between CHM and Western Medicine

Chinese herbal medicine is characterized by “Holistic regulation,” in which the organism is considered as a whole. With the equilibrium of the human body as the guidelines, practitioners of CHM pay more attention to the diseased patients rather than the suffered diseases itself. Particular western medicine always has a strong effect on a specific disease with focusing on a specific physiological target and ignoring the specific characteristics of each patient.

“Syndrome Differentiation based treatment” is another feature of traditional Chinese medicine, which means diagnosis and treatment based on an overall analysis of the illness and practitioners timely modify formulae in accordance with the varying syndromes and clinical manifestations. Thus, it is an individualized treatment for different patients even with the same disease. Particular western medicine usually works against a specific pathological process of diseases rather than a patient.

Many CHM and foods originate from the same source and the feature of “Homology of medicine and food” reflects that CHM has few adverse effects. Traditionally, some foods are preferred to drugs for health care, and certain kinds of herbs are regarded as foods. Hawthorn fruit and garlic are such examples. However, due to the strong pertinence, western medicine will inevitably cause side effects. Although the efficacy of CHM is not as strong as chemicals on a pathological process of CVDs, the feature of individualized intervention with minimum adverse effects makes CHM would be a good choice and will continue to make great contributions to the health of CVDs patients.

## Application of CHM in CVDs

Many experimental studies indicate that a great many herbs have potential benefits for CVDs. Herbal plant-based formulations or drugs are pivotal to traditional practices and application of plant products as CHM treatment has been widely used in patients with hypertension, atherosclerosis, congestive heart failure, angina pectoris and other cardiovascular risk factors.

### Application of Plant Products in Hypertension

Hypertension is a worldwide health problem with high morbidity and a major player in the onset of diseases such as atherosclerosis, stoke, peripheral artery disease, heart failure and coronary artery disease. Hypertension is defined as having a systolic BP of ≥140 mmHg and a diastolic BP of ≥90 mmHg. Every 20/10 (systolic BP/diastolic BP) mmHg increase indicates a higher risk stage of hypertension, stage 1 (140–159/90–99 mmHg) and stage 2 (≥160/100 mmHg) with the latter stage requiring immediate medical attention.

Notably, there are some formulae, many well known herbal products and their extracts or secondary metabolites of the herbs and spices exhibit antihypertensive effects. Interestingly, in a population-based database enrolling 984 CHM users and 2434 non-CHM users with hypertension among type 2 diabetes patients, the CHM users were characterized with slightly longer duration time from diabetes to hypertension, suggesting that CHM may restrain hypertension pathogenesis among type 2 diabetes patients (Lin et al., 2015). Here, we present the most commonly used formulae and a comprehensive alphabetical list of plant products with evidence suggesting beneficial in hypertension therapy.

#### Formulae

Yiqi Huaju Formula and Bushen Qinggan Formula are regularly applied in clinical practice for treating hypertension. Yiqi Huaju Formula consists of *Gastrodia elata, Uncaria, Eucommia bark, radix Scutellariae*, and *bitter butyl tea*. In a study with 43 hypertensive patients coupled with metabolic syndrome, 12 weeks of additional use of Yiqi Huaju Formula (together with anti-hypertensive drugs) significantly decreased systolic BP and 24-h BP variability compared with the control group (anti-hypertensive drugs plus placebo), indicating that Yiqi Huaju treatment may be better than antihypertensive drugs alone (Chen et al., 2013). Bushen Qinggan Formula consists of *Astragali Mongholici, Rhizoma Coptidis, Pollen Typhae, Rhizoma Alismatis*, and *Artemisiae Capillaries*. A favorable effect of Bushen Qinggan Formula was observed on control of BP variability level after 8 weeks of treatment in a randomized controlled pilot clinical trial (150 patients with hypertension). This was accompanied with decreased endothelin and elevated nitric oxide (NO)/endothelin in circulation, indicating improved endothelial function (Wu et al., 2014). In addition, according to the Guidelines for Diagnosis and Treatment of Common Internal Diseases in Chinese Medicine, there are some frequently used formulae for hypertension in clinic which are based on syndrome differentiation (China Association of Chinese Medicine, 2008). The formulae are listed in **Table 1**.

**Table 1:** Formulae for hypertension based on syndrome differentiation.

Symptom analysis	Therapeutic principle	Formulae	Ingredients	Chinese patent drug
Gan Huo Shang Yan	Qing Gan Xie Huo	Long-Dan-Xie-Gan Tang	Longdancao, Chaihu, Zexie, Cheqianzi, Sheng Dihuang, Danggui, Zhizi, Huangqin, Gancao.	Xie Qing Wan, Dang Gui Long Hui Wan
Tan Shi Nei Zu	Hua Tan Qi Shi, He Wei Jiang Zhuo	Ban-Xia-Bai-Shu-Tian-Ma Tang	Banxia, Baishu, Tianma, Chenpi, Fuling, Gancao, Gouteng, Zhenzhumu, Yujin.	Xuan Yun Ning Tablet
Yu Xue Nei Zu	Huo Xue Hua Yu	Tong-Qiao-Huo-Xue Tang	Dilong, Danggui, Chuanqiong, Chishao, Taoren, Honghua, Baizhi, Shichangpu, Laocong, Quanxie	Xin Mai Tong Tablet, Xin An Ning Tablet
Yin Xu Yang Kang	Ping Gan Qian Yang, Qing Huo Xi Feng	Tian-Ma-Gou-Teng Yin	Tianma, Gouteng, Shijueming, Niuqi, Duzhong, Sangjisheng, Huangqin, Zhizi, Fushen, Yejiaoteng, Yimucao	Qing Nao Jiang Ya Tablet, Nao Li Qing Capsule
Shen Jing Bu Zu	Zi Yang Gan Shen, Yi Jing Tian Sui	Zuo-Gui Wan	Shu Dihuang, Shanzhuyu, Shanyao, Guijia, Lujiaojiao, Gouqizi, Tusizi, Niuqi	Jian Nao Bu Shen Wan, Yi Ling Jing
Qi Xue Liang Xu	Bu Yi Qi Xue, Tiao Yang Xin Pi	Gui-Pi Tang	Dangshen, Baishu, Huangqi, Danggui, Longyanrou, Dazao, Fushen, Yuanzhi, Suanzaoren	N/A
Chong Ren Shi Tiao	Tiao She Chong Ren	Er-Xian Tang	Xianmao, Xianlingpi, Danggui, Bajitian, Huangbai, Zhimu, Baishao, Danshen, Yimucao, Cheqianzi.	Lu Gui Bu Shen Capsule

*Formulae should be modified according to specific syndromes and clinical manifestations.*

#### Natural Plant Products

##### Allium sativum L (Amaryllidaceae)

*Allium sativum* L is also called garlic. It receives emerging interest from pharmacologists and health practitioners with its multi therapeutic effects, including hypotensive capacity, anti-inflammatory, antioxidant, antibacterial and hypocholesteremic properties. Among the garlic’s organo-sulfur constituents, allicin is the most responsible one. Fresh garlic directly releases allicin while chewing, whereas dried garlic preparations lack allicin but contain both allinin and allinase which could convert allinin to allicin. Evidence from many investigations and meta-analysis of randomized, controlled trials concluded that garlic supplements induce significant reduction in mean arterial pressure, drop in either systolic BP or diastolic BP with different extent. Interestingly, some studies indicate that aged garlic extract produces consistent lowering of BP compared to other forms of garlic (Blesken, 1992). However, more experiments are needed to confirm garlic’s effect on hypertension since some studies come with no conclusive result (Shao et al., 2016). Despite having these multifarious effects, garlic also produces a few minor side effects, including abdominal swelling, heartburn, flatulence, and acid reflux et al.

##### Andrographis paniculata (Acanthaceae)

*Andrographis paniculata* is a plant that is commonly known as the King of Bitter. Several hypotensive labdane-type diterpenoid compounds, andrographolide, 14-deoxy- 11,12-didehydroandrographolide and 14-deoxyandrographolide have been identified in *Andrographis paniculata* extracts. Animal studies show that treatment with its extracts decrease angiotensin converting enzyme and ROS activities in SHRs leading to decrease in BP (Zhang and Tan, 1996). *Ex vivo* studies demonstrate that the extracts reduce vascular resistance reflected by decreased coronary perfusion pressure in rat isolated hearts (Awang et al., 2012). However, no clinic trials have yet been conducted using the King of bitter.

##### Apium graveolens L (Apiaceae)

*Apium graveolen*s L is also known as celery. The hypotensive effect of celery has been reported in *in vivo* animal studies. The extracts of celery reduce BP in deoxycorticosterone acetate-induced hypertensive rats or animal model of SHR (Moghadam et al., 2013). Importantly, extracts and constituents of celery have been reported to lower arterial pressure in humans, possibly by lowering levels of circulating catecholamines and decreasing vascular resistance (Houston, 2005).

##### Bidens pilosa L (Compositae)

*Bidens pilosa* L is also called Beggar’s Tick. It is native to America and belongs to the family Compositae. Extracts of its leaves were able to prevent and attenuate high BP in different hypertensive rat models, both SHR and fructose-fed hypertensive rats (Steiner et al., 1996). In fructose-fed rats, 75 and 150 mg/kg of methanolic leaf extract of *B. pilosa*, showed both therapeutic and preventative effect on systolic BP (Steiner et al., 1996). However, clinical trials are required to determine the potential effect of this plant on hypertension.

##### Camellia sinensis (Theaceae)

*Camellia sinensis*, also known as tea, is most frequently consumed and only second to water. Its cardiovascular protective effect is well known worldwide. Catechins, the major flavonoids in tea, include (–)- epicatechin, (–)-epicatechin-3-gallate, (–)- epigallocatechin, and (–)-epigallocatechin-3-gallate. (–)-epigallocatechin-3-gallate constitutes the primary component of tea’s total catechins. Although some trials come to conclusion of no change in BP subsequent to drinking tea, lots of studies suggest that tea consumption reduces both systolic BP and diastolic BP by ∼2 mmHg each. Interestingly, the green tea evokes a more powerful hypotensive effect compared to black tea.

##### Coptis chinensis (Ranunculaceae)

It’s commonly known as goldthread and widely used in Chinese folk medicine. The goldthread and its main component berberine, have the ability to lower BP. Indeed, a recent meta-analysis of twenty-seven randomized controlled trials involving 2569 patients reported that berberine can cause a significant hypotensive effect (Lan et al., 2015). In addition, this meta-analysis concluded that combined with an oral hypotensor, berberine can significantly reduce BP more than the hypotensor alone can do. The magnitude of the decrease was at an average of 4.91 and 2 mmHg for systolic BP and diastolic BP, respectively (Lan et al., 2015).

##### Crataegus spp. (Rosaceae)

It is also known as Hawthorn or Shanzha. The plants are shrubs that belong to a genus comprising almost 300 species and have been used in traditional medicine for treatment of CVDs since the seventeenth century. Hawthorn is used to dissipate food accumulation, to improve blood circulation, and to disperse blood stasis. The antihypertensive actions should be credited to the plant’s multiple components: flavonoids (hyperoside, quercetin, rutin, and vitexin) and oligomeric proanthocyanidins (OPCs, epicatechin, procyanidin, and procyanidin B-2). Modest decreases in BP have been observed in a few human-based studies with a demographic of hypertensive patients (Walker et al., 2002; Tassell et al., 2010). In a randomized, double-blind, placebo-controlled study where mildly hypertensive subjects were treated with 500 mg of hawthorn extract for 10 weeks, a promising tendency for a reduction in diastolic BP was reported (Walker et al., 2002). In another clinic study, administration of hydro-alcoholic extracts of hawthorn flowers to hypertensive patients (age range 40–60 years) for 3 months induced a decrease in both systolic BP and diastolic BP by around 13 and 8 mmHg, respectively (Asgary et al., 2004).

##### Crocus sativus L (Iridaceae)

*Crocus sativus* L, also known as Saffron, a plant indigenous to Southwest Asia, Spain, Greece, and Morocco, is a stemless herb. Saffron’s main components include crocin, picrocrocin, safranal, and crocetin. Several reports support the use of saffron for anti-hypertensive benefits. A clinical study reports that 400 mg of Saffron tablets administered for 7 days are able to significantly reduce the systolic BP and mean arterial pressure in healthy humans by 11 and 5 mmHg, respectively (Modaghegh et al., 2008). Six weeks old stroke-prone SHRs are given crocetin for 3 weeks and this treatment significantly moderates the increase in systolic BP observed with age (Higashino et al., 2014). Saffron also demonstrates vasorelaxant activities in different animal models, including male Sprague-Dawley rats (Fatehi et al., 2003), stroke-prone SHRs (Higashino et al., 2014), deoxycorticosterone acetate-salt induced hypertensive male Wistar rats (Imenshahidi et al., 2010) but not normotensive rats (Imenshahidi et al., 2015).

##### Hibiscus sabdariffa L (Malvaceae)

Also known as Roselle, *Hibiscus sabdariffa* is widely used for hypertension, fever, and other diseases in folk medicine. Different parts of this plant (buds, calyx, flowers, leaves, and petals—fresh or dried) are used for health purposes and as refreshing beverages, food items (jams, preserves), or lotions. Hibiscus’s effects of hypotension have been extensively investigated in both animal and human studies (McKay et al., 2010; Ojeda et al., 2010; Inuwa et al., 2012). The blood lowering effects were notable subsequent to treatment with dried extract of Roselle calyx for 4 weeks in patients with stage 1 or 2 hypertension (Herrera-Arellano et al., 2007). In addition, consuming Roselle’s tea (240 ml—three times a day for 6 weeks) significantly reduces systolic BP, diastolic BP, and mean arterial pressure by 7.2, 3.1, and 4.5 mmHg, respectively in mild and pre-hypertensive patients (65 subjects, age range 30–70 years old) (McKay et al., 2010), supporting Hibiscus’ therapeutic role in ameliorating hypertension.

##### Hippophae rhamnoides L (Elaegnaceae)

*Hippophae rhamnoides* L is also called Saji or Sea buckthorn and its dry fruits are used in China as an herbal medicine. Analysis showed that the powder made of dry hippophae fruits contains the vitamins C, B1, B2 and E, provitamin A, rutin, serotonin, cytosterol, selenium and zinc. The stroke-prone SHR were fed *ad libitum* with blocks of rat chow supplemented with Hippophae powder and the effect of lowering BP was examined. The mean arterial BP and heart rate were significantly decreased by the Hippophae treatment (Koyama et al., 2009), which is in accord with previous studies showing that Hippophae extracts decreased intracellular Ca^2+^ in cultured VSMCs (Zhu et al., 2005).

##### Nigella sativa L (Ranunculaceae)

*Nigella sativa* L, also known as Black Cumin or Habbatul barakah (seed of blessing), has been used in the kitchens of Europe, the Middle East, Africa, and Asia for centuries. Thymoquinone, one of the most abundant and bioactive components, has been identified as the major element in its healing effects. Similar to other herbs, *N. sativa* and its constituents have been shown to reduce BP in humans and different animal models of hypertension. *N. sativa*’s seed extract administrated orally (either 100 or 200 mg, two times per 24 h for 8 weeks) to mild hypertensive male patients recorded a dose-dependent fall in both systolic BP and diastolic BP (Ried, 2016). Again, in a randomized, placebo-controlled, double-blind study with 70 healthy subjects, *N. sativa* oil caused a significant decrease of 10.6 mmHg in systolic BP and 9.6 mmHg in diastolic BP (Fallah Huseini et al., 2013).

##### Panax ginseng C.A. Mey (Araliaceae)

*Panax ginseng*, also called Panax or Renshen, has been used in folk medicine for several centuries. Ginseng is prepared and administered in various forms, either as a solid: tablets, capsules, dried roots; or as a liquid: oil, extracts or tea. There are four most common species of Ginseng, including *P. ginseng* (Asian or Korean Ginseng), *P. quinquefolius* (American Ginseng), *P. japonicas* (Japanese Ginseng) and *P. notoginseng* (Chinese Ginseng) are the four most common species of ginseng. Heterogeneous triterpenoid saponins and steroid glycosides or ginsenosides (or panaxosides) are the active principle components of ginseng. Several clinical trials have been conducted to assess the efficacy of ginseng in BP regulation. Both Asian/Korean Ginseng extract and American Ginseng caused a significant decrease in systolic BP and diastolic BP in hypertensive patients (Mucalo et al., 2013; Rhee et al., 2014). The mode of action may be related with antagonizing a calcium ion channel in vascular tissues, which may result in lowering BP. However, conflicting reports of elevated BP also exist (Jang et al., 2011; Kim, 2012). For example, studies have shown that low doses of ginseng raise BP, while higher concentrations are hypotensive (Jang et al., 2011), which need more experiments to clarify the differentiation.

##### Pueraria lobata (Willd) ohwi (Leguminosae)

It is also known as Kudzu root or Gegen. Kudzu root has traditionally been used in Chinese medicine for treating CVDs and Type II diabetes. It is a rich source of polyphenolic compounds, including isoflavones, isoflavonoid glycosides, coumarins, puerarols, but-2-enolides and their derivatives. Puerarols attract more attention to investigate the direct effect on BP. According to Wong’s review, the anti-hypertensive effect of puerarin was shown as early as 1980s evidenced by reduced BP, heart rate and plasma rennin activity in SHRs and this effect may be exerted through blocking the effect of β-adrenergic receptors (Wong et al., 2011). Verapamil is a positive control inhibiting Angiotensin II type I receptor and angiotensin-converting enzyme 2 in SHRs. Similar to it, puerarin treatment significantly repressed the mRNA levels of Angiotensin II type I receptor and angiotensin-converting enzyme 2 in cardiac tissues (Ye et al., 2008). Further interdisciplinary collaboration to bridge the gap between traditional medicine and modern biomedical medicine is needed for the development of Kudzu root as an effective medicine for the management of hypertension and other CVDs.

##### Salviae miltiorrhizae (Lamiaceae)

It is also known as Danshen, or red/Chinese sage. Danshen is one of the oldest and most frequently consumed Chinese traditional herbs and is commonly used for the treatment of CVDs. Sal A, Sal B, Danshensu, and tanshinones are its most effective ingredients. A combination treatment of Danshen and Gegen was shown to lower BP in SHRs and to induce relaxation of several kinds of arteries including porcine coronary arteries, rat aorta, and basilar arteries (Al Disi et al., 2015). Apart from its vasodilatory capacity, Danshen expresses additional anti-hypertensive parameters such as antioxidative, anti-proliferative, and anti-inflammatory activities which will be discussed in more details in Section “Mechanisms of Plant Products in the Attenuation of CVDs.”

##### Scutellaria baicalensis Georgi (Lamiaceae)

The dried roots of *S. baicalensis*, also known as Huangqin in China, have been widely employed in traditional CHM as popular antibacterial and antiviral agents. Baicalein and its glycoside, baicalin, are the major ingredients responsible for its beneficial effects. Its BP lowering effect in dog and rat was reported as early as 1980’s (Huang et al., 2005). However, the baicalein exerts a complex effect on the agonist contracted rat mesenteric arteries. Within a relative low concentration range (0.3–10 μM), baicalein potentiates vasoconstricting responses to phenylephrine, U46619, or to elevated extracellular potassium in endothelium-intact artery rings (Chen et al., 1999; Tsang et al., 2000), whilst it produces relaxations at concentrations greater than 10 μM (Chen et al., 1999). Since the increased vessel contraction is absent upon removal of the endothelium while the relaxant effect of baicalein remains (Chen et al., 1999), endothelium-derived vasoactive factors may be involved in the bidirectional effect on vascular function. To be specific, baicalein and baicalin increase the evoked contractile responses likely through inhibition of NO production and/or release in the endothelium (Huang et al., 2000, 2002; Tsang et al., 2000). The mechanisms by which baicalein lowers BP will be discussed in Section “Regulating Calcium Levels in PKA/PKG/PKC-Dependent Way.”

### Application of Plant Products in Atherosclerosis and Dyslipidemia

Atherosclerosis, one of the primary causes of CVDs, is a vascular disease that occurs at susceptible sites in major arteries. It is an inflammatory process and ultimately causes stenosis or thrombosis with potentially lethal distal ischemia. The main elements involved in the complex pathogenesis of atherosclerosis include hyperlipidemia, endothelial injury, LDL subendothelial retention and oxidation, monocyte migration and activation, VSMC migration and proliferation, foam cell formation, apoptosis and efferocytosis and unresolved inflammation. Among them, dyslipidemia is the primary independent risk factors, which is characterized by elevated level of total cholesterol (TC), triglyceride (TG), LDL-C and by lowered level of high-density lipoprotein cholesterol (HDL-C) in serum.

The description of the clinical manifestations and treatment of atherosclerosis can be found in the classic traditional Chinese medicine book Inner Canon of yellow Emperor, as early as 500BC. Atherosclerosis, dyslipidemia and its resulting heart disease have been treated with numerous herbal remedies for centuries. Then, we present a comprehensive alphabetical list of plant products which are most commonly used to attenuate atherosclerosis and lower hyperlipidemia.

#### *Allium fistulosum* L (Amaryllidaceae)

*Allium fistulosum* L is widely cultivated in Southern China. Treatment with Fistular onion stalk, the derivant from *A. fistulosum*., induces a significant reduction in the average lesion area of atherosclerosis and preservation of the vascular wall and immune cell infiltration. The extract also reduces the levels of the inflammatory cytokines IL-1β, IL-6, MCP1 and TNFα and downregulated the local activity of the rennin-angiotensin-aldosterone system in the aortic tissue. In addition, extract treatment inhibits several local inflammatory signaling pathways by preventing its activation, including phosphorylation of the NFκB, Janus kinase/signal transducers and activators of transcription and mitogen-activated protein kinase pathways (He B. et al., 2014). These data indicate that fistular onion stalk extract may be useful for the attenuation of atherosclerosis, and the mechanism includes the regulation of the local inflammatory responses.

#### *Allium sativum* L (Amaryllidaceae)

In addition to the anti-hypertensive effect, there is also wide spread belief among general public that *Allium sativum* L (garlic) has beneficial effects on dyslipidemia in patients. Seventy type 2 diabetes patients with newly diagnosed dyslipidemia were divided into two groups and were given tablet garlic 300 mg (containing 1.3% allicin) twice daily and identical placebo tablets respectively. 12-week of garlic treatment induces a significant reduction in TC and LDL-C, a significant increase in HDL-C (Ashraf et al., 2005). In a double-blind, crossover study of moderately hypercholesterolemic men, treatment with 7.2 g aged garlic extract induced a maximal decrease of 6.1% in serum TC levels and 4.6% in LDL cholesterol levels (Steiner et al., 1996). These effects were Although one recent meta-analysis concluded that garlic decreases TC to a modest extent, an effect driven mostly by the modest decreases in TG, with no appreciable effect on LDL or HDL cholesterol (Reinhart et al., 2009), many studies suggest garlic to be effective in improving lipid metabolism (Ried, 2016). Since some meta-analysis has been based on trials with inadequate study designs, methodological deficiencies, the effect of garlic on atherosclerosis and dyslipidemia awaits further investigation.

#### *Astragalus propinquus* (Fabaceae)

The dried root of *Astragalus propinquus*, Radix Astragali, is also known as Huangqi. Polysaccharides, flavonoids, and sponins are the main active components of membranous milkvetch root. Studies have shown that Huangqi extract significantly decreased the area of atherosclerosis plaques (17.24% ± 4.22% vs. 49.87% ± 9.37%, *P* < 0.01) and level of serum oxLDL (5.2 ± 6.1 μg/ml vs. 15.8 ± 5.4 μg/ml, *P* < 0.01) in ApoE^-/-^ mice (You et al., 2012). Effects of its extract and components on lipid profile are also summarized in a review, in which the serum of TG, TC, and LDL-C levels were reduced by both *Astragalus mongholicus* extracts treatment at 0.4 and 0.8% for 5 weeks and polysaccharides from *Astragalus* administered at an oral dosage of 40 mg, 100 mg/kg/day in hyperlipidemia rats for 40 days (Guo et al., 2014). Accordingly, the HDL-C levels were increased by these treatments (Guo et al., 2014).

#### *Coptis chinensis* Franch. (Ranunculaceae)

Rhizoma Coptidis, also known as Huanglian, is derived from the dried root and rhizome of *Coptis chinensis* Franch., *Coptis deltoidea* C. Y. Cheng et Hsiao, and *Coptis teeta* Wall. Its main components include lignans and alkaloid, in which berberine is the active component for lipid lowering. Because of berberine’s active effect for lipid lowering, most of the studies focus on this component. Studies have shown that berberine administration significantly decreased the serum TG, TC, LDL-C, when taken orally or injected intraperitoneally (Zhou et al., 2008; Chang et al., 2012). In addition, Caucasian obese human subjects were orally given 500 mg berberine, three times a day for 12 weeks. The blood lipid was significantly reduced and TG, TC were decreased by 23 and 12.2%, respectively (Hu et al., 2012). In a meta-analysis, berberine induced a significant reduction in these biomarkers in 874 participants in11 randomized controlled trials (Ma et al., 2013).

#### *Crataegus* spp. (Rosaceae)

Known as hawthorn, its hypolipidemia effect has been investigated widely in animal studies. In high-fat diets fed mice, the oral administration of the whole extract at a dosage of 250 mg/kg/day for 7 days induced blood lipid decrement (Niu et al., 2011). Then, the specific effects of aqueous and ethanol extracts of hawthorn on lipid profiles were compared. In a high-fat emulsion fed mice, both ethanol and aqueous extracts possessed hypolipidemia activities and the ethanol extract exhibited more favorable effects than the aqueous extract (Shao et al., 2016). This lipid lowering effect of hawthorn mostly contributes to inhibition of the progression of atherosclerosis which was evidenced by the significantly inhibited pathological changes and reduced intima-media thickness in the arteries (Zhang et al., 2013). In-depth exploration demonstrated that the lipid-lowering action may be due to the anti-inflammation activities, the upregulation of PPARα to facilitate β-oxidation-related enzymes in liver leading to lipid degradation, enhanced expression of hepatic LDL receptors resulting in a greater influx of plasma cholesterol into the liver, and the suppressed cholesterol biosynthesis and increased degradation of cholesterol to bile acids (Niu et al., 2011; Walden and Tomlinson, 2011; Zhang et al., 2013; Shao et al., 2016). The hypolipidemic effects of hawthorn in clinic remain further study.

#### *Epimedium brevicornum* Maxim (Berberidaceae)

*Epimedium brevicornum* Maxim, an important traditional CHM, has been used widely for 1000s of years in China, Korea, and Japan. Icariin, a flavonoid isolated from *Epimedium brevicornum* Maxim, is considered as the main pharmacological active constituent. In the high cholesterol diet-induced atherosclerosis rats, the levels of blood lipids including TC, TG, LDL-C, and malonaldehyde were significantly increased, while HDL-C and SOD were significantly decreased. Icariin succeeded in improving these biochemical parameters toward the normal values (Hu et al., 2016).

#### *Fallopia multiflora* Thunb. (Polygonaceae)

Its dried root tuber is Radix Polygoni Multiflori, also known as Heshouwu. Raw and prepared pharmaceutical forms are frequently used. Guo et al. (2014) reviewed the hypolipidemic effect of Heshouwu from some Chinese literature which demonstrated that the total extract of the Radix Polygoni Multiflori significantly reduced the serum levels of TC, TG and LDL-C in hyperlipidemic rats when administered at an oral dosage of 12 mg and 24 mg/kg/day for 4 weeks. Similarly, these lipid profiles in the hyperlipidemic rats were lowered by the treatment of the ethlacetate extracting fraction and stilbene glycoside from the tube of Radix Polygonum Multiflorum too (Guo et al., 2014).

#### *Fermentum Rubrum* (Aspergillaceae)

Fermentum Rubrum, popularly known asred yeast rice, is the fermented product of *Monascus purpureus* on rice. It is composed of 13 kinds of natural statins, unsaturated fatty acids, ergosterol, amino acids, flavonoids, alkaloid, trace element, and so forth. Some clinic studies have demonstrated the lipid lowering effects of the red yeast rice. A twice daily dose of red yeast rice at 600 mg for 8 weeks was found to reduce LDL-C by 27.7%, TG by 21.5%, and TC by 15.8% (Lin et al., 2005). Similar effects were observed in 72 patients with idiopathic persistent nephritic syndrome with secondary dyslipidemia (Gheith et al., 2008, 2009). Xuezhikang capsule is the extract of red yeast rice. Scholars in China made a systematic review on the clinical randomized controlled trials for hyperlipidemia treatment with Xuezhikang, which included a total of 6520 participants in 22 randomized trials. It was concluded that Xuezhikang remarkably lowered TC, TG, and LDL-C compared with the inositol nicotinate (Fallah Huseini et al., 2013).

#### *Olea europaea* L. (Oleaceae)

The beneficial effects of *Olea europaeal* L., especially olive leaf extract are known since antiquity, with numerous records confirming its therapeutic use. According to the European Pharmacopeia, the most abundant substances in standardized dry leaf extracts are oleuropein, hydroxytyrosol, caffeic acid, tyrosol, apigenin, and verbascoside, with oleuropein being the major component of olive leaf extract. Olive leaf extract treatment reduces the atherosclerostic lesions size and the thickness of intima in atherosclerotic rabbits, accompanied with decreased levels of atherosclerotic markers, including serum TG, TC, LDL, HDL, and malonaldehyde with a parallel downregulation of MCP1, vascular cell adhesion molecule-1, NFkB and TNFα (Wang et al., 2008). Consistent with it, another animal study showed that a 1-month intake of hydroxytyrosol by rabbits consuming an atherogenic diet results in a reduced size of atherosclerotic lesions compared to control animals (Gonzalez-Santiago et al., 2006). However, the results originating from *in vivo* models are divergent. The intake of hydroxytyrosol in an *in vivo* apo-E knockout mice model showed that this compound, when administered for 10 weeks, leads to atherosclerotic lesions associated with the activation of monocytes and modification of the blood lipid profile (Acin et al., 2006). These studies indicate that under certain conditions the phenol could be rather harmful than cytoprotective.

#### *Panax ginseng* C.A. Mey (Araliaceae)

The resources and components have been discussed in Section “Application of Plant Products in Hypertension.” A serious of studies has demonstrated the hypolipidemic effects of its components. For example, the levels of plasma TC, TG, and LDL-c in C57/BL-ApoE gene knockout hyperlipidemia rats were reduced by intragastric administration of ginseng saponins at an oral dosage of 2 mg/kg/day for 90 days. Ginseng saponin is divided into Rb1, Rb2, RC, Rd, Re, and Rl. Ginseng saponin Rb administered at an oral dosage of 50–200 mg/kg/day in hyperlipidemia rat for 12 days significantly reduce the TG, TC, and LDL-c levels in serum and liver (Yue et al., 2012). Ginseng radix can be metabolized into compound k in small intestinal. *In vitro*, the metabolites significantly activate the AMP-activated protein kinase (AMPK) to affect the lipid metabolism in insulin-resistant HepG2 human hepatoma cells (Kim et al., 2009).

#### *Pueraria lobata* (Willd) *ohwi* (Leguminosae)

Also known as Kudzu Root, its effects on hyperlipidemia are investigated in a series of studies. Puerarin, the active component significantly reduced the serum lipid concentration or hepatic cholesterol levels in hyperlipidemia rats, when administrated at oral dosages of 300 mg/kg/day for 4 weeks or injected intraperitoneally at 50 mg/kg/day for 30 days (Yan et al., 2006). Furthermore, hepatic lipid metabolism was also enhanced in ovariectomized rats by oral administration of Kudzu root (Wang et al., 2004).

#### *Reynoutria japonica* Houtt (*Polygonaceae*)

*Reynoutria japonica* Houtt is a synonym of Polygonum cuspidatum siebold and Zucc. Its dried root and rhizome is known as Huzhang in Chinese medicine. Polydatin and resveratrol are the primary active components. It has been found that oral administration of polydatin at dosages of 25 mg–150 mg/kg/day for 15–21 days significantly decreased TC, TG, and LDL-C levels or TC/HDL-C ratio in hyperlipidemic hamsters or rabbits (Du et al., 2009; Xing et al., 2009). Intragastrical administration of another component resveratrol at dosages of 10–70 mg/kg/day for 6 weeks not only decreased levels of serum lipid but also attenuated diet-induced hepatic steatosis and atherosclerosis index in hyperlipidemic mouse (Xie et al., 2014).

#### *Rheum palmatum* Linn (Polygonaceae)

*Rheum palmatum* Linn., *Rheum tanguticum* Maxim. Ex Balf., or *Rheum officinale* Baill belongs to the family of Poygonaceae. Radix Et. Rhizoma Rhei, also known as Dahuang or rhubarb, is derived from the root and rhizome of Polygonaceae members. The main active component includes rhein, aloe emodin, emodin, chrysophanol, and physcion. There is a series of studies exhibited the lipid-lowering effects in animals or patients. For example, the decoction boiled from rhubarb at 8, 16, and 32 g/kg/day for 5 days caused fatty degeneration of the hepatic cell in mice. Administration of the powders of rhubarb, at 5 g/day orally for 24 weeks, decreased serum TG and TC levels in patients with diabetic nephropathy (Gao Q. et al., 2010). Rhein, one of the active components or rhubarb, at an oral dosage of 150 mg/kg/day for 12 weeks, was proved to be lowering serum TG, TC, and LDL-C levels in mice with diabetic nephropathy. Thus the lipid-lowering effect of rhubarb, at least, partially dues to the component rhein. Danthron is another extract of rhubarb. It was observed that danthron dose-dependently promoted the phosphorylation of AMPK and acetyl-CoA carboxylase in both HepG2 and C2C12 cells. Meanwhile, danthron significantly reduced sterol regulatory element-binding protein 1c synthesis and fatty acid synthase gene expressions, contributing to the inhibitory effect on lipid metabolism (Han et al., 2013). However, some side-effects, including vomiting, headache, diarrhea and abdominal pain, are reported by some volunteers.

#### *Salviae miltiorrhizae* (Lamiaceae)

It has been described in Section “Application of Plant Products in Hypertension.” It is widely used to treat patients with coronary artery disease in China. Studies have showed decrement in levels of TC, TG and LDL-C when administrated with its aqueous extracts orally (50, 100, and 150 mg/kg/day for 4 weeks in hyperlipidemic rats) (Ji and Gong, 2008) or intravenous injected with tanshinone IIA, the main component (80 mg/day for 14 days in patients with diabetes) (Jang et al., 2008).

#### *Scutellaria baicalensis* Georgi (Lamiaceae)

*Scutellaria baicalensis*, known as Huangqin, is derived from the dried root of *Scutellaria baicalensis* Georgi. The most effective lipid-lowering component is flavonoid compound. Both stem-leaf total flavonoids administrated at oral dosages (25–150 mg/kg/day) and 0.05% radix extract added to the diet significantly reduced the serum TG, TC, and LDL-C levels or increased HDL-C levels (Regulska-Ilow et al., 2004). In addition, treatment with flavonoids from the Huangqin leaves increased the activity of lecithin cholesterol acyltransferase in hyperlipidemia rats (Regulska-Ilow et al., 2004), contributing to the lipid-lowering effect.

#### *Senna obtusifolia* Linn. (Fabaceae) or *Cassia tora* Linn. (Leguminosae)

The ripe seed of *Senna obtusifolia* Linn. (Fabaceae) or *Cassia tora* Linn. (Leguminosae) is Semen Cassia, known as Juemingzi. Anthraquinone glycosides are the active component. It has been found that the extract from Semen Cassia at lower doses (8–15 mg/kg/day) for 35 days, or at higher doses (180 mg/kg/day) for 7 days, significantly decreased TC, TG and LDL-C and increased HDL-C in hyperlipidemic animal models. Similar results were induced by administration with the total anthraquinone from Semen Cassia for 2 months in alcohol treated SD rats (Guo et al., 2014).

#### The Others

There are many other herbs which show the hypolipidemic effects and widely used in Chinese medicine. These herbs include Rhizoma (Chuanxiong), Rhizoma Curcumae Longae (Jianghuang), Rhizoma Alismatis (Zexie), Semen Plantaginis (Cheqianzi), and Folium Nelumbinis (Heye). The oral administration of the extracts or the active components exerts the hypolipidemic effects and improving lipid metabolism (Guo et al., 2014). In addition, there are some formulae for dyslipidemia which are applied in clinic basing on syndrome differentiation (China Association of Chinese Medicine, 2008). The formulae are listed in **Table 2**.

**Table 2:** Formulae for dyslipidemia based on syndrome differentiation.

Symptom analysis	Therapeutic Principle	Formulae	Ingredients	Chinese patent drug	Homo remedy
Wen Re Yun Jie	Qing Re Li Shi	Long-Dan-Xie-Gan Tang	Longdancao, Huangqin, Sheng Dihuang, Zhizi, Chuanmutong, Chaihu, Danggui, Cheqianzi, Zexie	Sang Ge Jiang Zhi Wan	Jue Ming Zi, Zexie, Shanzha Slice
Tan Shi Nei Zu	Hua Tan Qi Shi	Wen-Dan Tang	Chenpi, Banxia, Fuling, Zhishi, Zhuru, Baishu, Dannanxing	Zhi Ke Qing Capsule, Xue Zhi Ling Tablet, Yue Jian Cao Oil-emulsion	
Tan Yu Jie Zhi	Hua Tan Xing Yu	Er-Chen-Tang and Xue-Fu-Zhu-Yu Tang	Chenpi, Baixia, Fuling, Chaihu, Zhishi, Chi Baishao, Sheng Dihuang, Danggui, Chuanqiong, Taoren, Honghua	Shan Zhuang Jiang Zhi Tablet, Tong Mai Jiang Zhi Tablet	
Pi Xu Shi Sheng	Jiang Pi Li Shi	Wei-Ling Tang	Chenpi, Houpu, Zisuye, Fuling, Cangshu, Zhuling, Cheqianzi, Zexie	Jian Pi Jiang Zhi Granule, Zhi Bi Tuo Capsule, Jiao Gu Lan Zong Gan Tablet	
Gan Shen Yin Xu	Zi Bu Gan Shen	Yi-Guan-Jian and Qi-Ju-Di-Huang Wan	Danggui, Sheng Dihuang, Gouqizi, Shanyao, Fuling, Shanzhuyu, Zexie, Mudanpi, Juhua, Beishashen	Jiang Zhi Ling Tablet, Yu Jin Fang Capsule, Zhi He Shou Wu Granule	
Pi Shen Yang Xu	Bu Shen Jian Pi	You-Gui-Wan and Shen-Ling-Bai-Shu San	Shu Dihuang, Gouqizi, Duzhong, Tusizi, Fuzi, Rougui, Shanyao, Baibiandou, Dangshen, Fuling, Baishu	Dan Tian Jiang Zhi Wan	

*Formulae should be modified according to specific syndromes and clinical manifestations.*

### Application of Plant Products in Heart Diseases

Cardiovascular diseases involving the heart includes angina, myocardial infarction, cardiomyopathy, congenital heart disease, and heart failure, etc. Some of the diseases interact on each other or on other vascular diseases. For example, coronary artery diseases involve atherosclerosis, which may be caused by hypertension, hyperlipidemia, whereas heart failure can arise as consequence of large myocardial infarctions. Thus, herbs applied in treatment of hypertension, atherosclerosis and lowering lipid profiles should contribute to attenuating heart diseases too. Here, we present formulae and the natural plants frequently used in clinic and lab studies in alphabetical order.

#### Formulae

There are some formulae which are frequently used to treat heart diseases. Chinese herbal formula Sini Tang/dicoction (*Aconitum carmichaelii, Zingiber officinale, Glycyrrhiza uralensis*, or/and *Cinnamomum cassia*) was reported to improve cardiac function (ejection fraction and fractional shortening) after myocardial infarction in rats. With ^1^H NMR and UHPLC-MS measurement, 21-day application of Sini decoction effectively reversed the urinary metabolic profiles in the myocardial infarction rat model (Tan et al., 2012; Sheu et al., 2014). Another formula consists of *Terminalia arjuna, Cactus grandiflorous, Crataegus oxyacantha*, and *Piper nigrum*. Preadministration and postadministration of this herbal mixture restore the levels of biomarker of cardiotoxicity, including cardiac marker enzymes, lipids profile, and antioxidant enzymes (Lin et al., 2011). Rhodiola formulation is claimed to relieve the symptoms of ischemic heart disease and improve the electrocardiography in a number of clinical studies (Yu et al., 2014). A meta analysis including 13 studies (*n* = 1672) demonstrated that the effectiveness of Rhodiola formulations was higher compared to control group (symptoms improvement: OR = 2.40, 95% CI: 1.57–3.66, *P* < 0.0001; electrocardiography improvement: OR = 1.48, 95% CI: 1.17–1.87, *P* < 0.01). In addition, the ORs of symptomatic and electrocardiography improvement in Rhodiola formulations versus other CHMs, versus routine western medicine, and Rhodiola formulations plus routine western medicine versus routine western medicine were 1.51, 2.64, 5.63, and 1.33, 3.11, 2.27, respectively (Yu et al., 2014). These findings indicate that the formulations have a positive effect on treating ischemic heart disease alone or in combination with routine western medicine. In addition, there are some formulae for angina pectoris or coronary heart disease or heart failure which are based on syndrome differentiation (China Association of Chinese Medicine, 2008) and are listed in **Tables 3** and **4**.

**Table 3:** Formulae for coronary heart disease and angina pectoris based on syndrome differentiation.

Symptom analysis	Therapeutic Principle	Formulae	Ingredients	Chinese patent drug	Homo remedy
Xin Xue Yu Zu	Huo Xue Hua Yu, Tong Luo Zhi Tong	Dan-Shen Yin or Tao-Hong-Si-Wu Tang	Taoren, Danshen, Chuanqiong, Chishao, Honghua, Sheng Dihuang, Sharen, Tanxiang	Fu Fang Dan Shen Tablet, Yin Xing Ye Capsule	Ren Shen San Qi San, Huo Xue Xin Tong San
Tan Zhuo Bi Zu	Hua Tan Xie Zhuo, Yi Bi Tong Yang	Guo-Lou-Xie-Bai-Ban-Xia Tang	Guolou, Xiebai, Fabanxia, Fuling, Chenpi, Zhishi, Xingren	Er Chen Wan	
Yin Han Ning Zhi	Wen Tong Xin Yang, Kai Bi San Jie	Guo-Lou-Xie-Bai-Gui-Zhi Tang	Guolou, Xiebai, Guizhi, Baijiu, Biba, Gaoliangjiang	Guan Xin Su He Wan, Su He Xiang Wan	
Qi Yin Liang Xu	Yi Qi Yang Yin, Tong Luo Zhi Tong	Sheng-Mai-San	Huangqi, Dangshen, Maidong, Wuweizi, Danshen, Honghua, Sanqifen	Sheng Mai Yin, Tian Wang Bu Xin Dan, Bu Xin Qi and Yang Xin Yin Oral Solution	
Xin Shen Yang Xu	Wen Bu Xin Shen	Jin-Kui-Shen-Qi Wan	Guizhi, Danfupian, Sheng Dihuang, Shanzhuyu, Mudanpi, Fuling, Zexie, Sanqifen	Jin Kui Shen Qi Wan, Gui Fu Li Zhong Wan	

*Formulae should be modified according to specific syndromes and clinical manifestations.*

**Table 4:** Formulae for heart failure based on syndrome differentiation.

Symptom analysis	Therapeutic Principle	Formulae	Ingredients	Chinese patent drug	Homo remedy
Xin Fei Qi Xu	Bu Yi Xin Fei	Bao-Yuan Tang and Bu-Fei Tang	Dangshen, Shu Dihuang, Ziwan, Sangbaipi, Rougui, Jiu Gancao, Wuweizi, Fuling, Huangqi, Suan Zaoren	Bu Xin Qi Oral Solution, Ren Shen Bao Fei Wan	Ting Li Zi, Fu Shou Cao
Qi Yin Liang Xu	Yi Qi Yang Yin	Wu-Wei-Zi Tang	Dangshen, Maidong, Wuweizi, Guizhi, Baishao, Sheng Dihuang, Ajiao, Jiu Gancao, Jiu Huangqi	Sheng Mai Yin	
Qi Xu Xue Yu	Yi Qi Huo Xue Li Shui	Bu-Yang-Huan-Wu Tang and Wu-Ling San	Huangqi, Danggui, Chishao, Dilong, Chuanqiong, Honghua, Taoren, Zexie, Baishu, Zhuling, Fuling, Guizhi	Xin Tong Oral Solution, Tong Xin Luo Capsule	
Yang Xu Shui Ting	Wen Yang Li Shui	Zhen-Wu Tang	Fuzi, Ganjiang, Niuqi, Chuanqiong, Chishao, Zexie, Baishu, Zhuling, Fuling	Bu Shen Kang Le Capsule, Xin Bao Wan	
Re Tan Yong Fei	Qing Fei Hua Tan, Xie Fei Li Shui	Qing-Jin-Hua-Tan Tang	Huangqin, Zhizi, Sangbaipi, Gualou, Maidong, Chuanbeifen, Jugeng, Gancao, Yiyiren, Dongguaren, Fuling, Yuxingcao	Qing Fei Hua Tan Wan, Xian Zhu Li Shui	
Han Tan Zu Fei	Wen Fei Hua Tan	Xiao-Qing-Long Tang and Ting-Li-Da-Zao-Xie-Fei Tang	Mahuang, Fabanxia, Chishao, Ganjiang, Guizhi, Wuweizi, Xixin, Tinglizi	Fu Fang Ge Qing Tablet, Ke Chuan Capsule	
Yin Jie Yang Tuo	Gu Yin Hui Yang Jiu Ni	Shen-Fu-Long-Mu Tang	Shengshaishen, Fuzi, Ganjiang, Maidong, Wuweizi, Longgu, Muli	Gu Shen Ding Chuan Wan, Ren Shen Gu Ben Wan	

*Formulae should be modified according to specific syndromes and clinical manifestations.*

##### Astragalus propinquus (Leguminosae, Fabaceae)

Injection of *Astragalus*, also known as Huangqi, is one of the most commonly used Chinese patent medicines for the treatment of chronic heart failure in China as complementary treatment to recommended Western therapies. Fu et al. (2011) has reviewed 62 clinic trials from 1205 articles. Although the available studies are not adequate to draw a conclusion on the efficacy of the Huangqi injection, it is observed to enhance myocardial contractility, improve circulation, and protect myocardial cells with modern pharmacological tools. For example, in an *in vivo* rat model of persistent myocardial ischemia produced by occlusion of the left anterior descending coronary artery, pretreatment of Huangqi extract significantly decreased the myocardial infarct size and the serum levels of lactate dehydrogenase, creatine kinase isoform MB, and cardiac troponin. Additionally, this treatment dramatically improved cardiac function, as assessed by dP/dt, left ventricular developed pressure, and left ventricular end-diastolic pressure (Ma et al., 2013).

##### Cecropia pachystachya (Urticaceae)

*Cecropia pachystachya*, popularly called ambay, is extensively used in herbal medicine of South America. *Cecropia pachystachya* Mart growing in a temperate region produced a positive inotropic effect on isolated rat hearts independent of beta-adrenergic effect. In contrast, the inotropic effect was prevented by pretreatment with high potassium media (stimulating Na-K-ATP pump), indicating an inhibition of the pump by ambay (Consolini et al., 2006).

##### Crataegus spp. (Rosaceae)

It has been described in previous sections (hawthorn). In isolated perfused hearts, extracts of hawthorn have been reported to show cardioprotective effects without affecting coronary blood flow (Nasa et al., 1993). Simultaneously, they demonstrated vasodilator actions and positive cardiac inotropic effect (Blesken, 1992). These results were verified in a multicenter, placebo-controlled, double-blind study, in which WS 1442, the special extract of hawthorn, improved cardiac function determined by heart rate product (systolic BP × heart rate) in patients with NYHA class II heart failure (Weikl et al., 1996). More studies are needed to investigate the mechanisms of hawthorn to attenuate CVDs.

##### Olea europaea L. (Oleaceae)

It has been described in previous sections and the main extract from the olive leaves is oleuropein. The rabbits were subjected to a 30 min period of regional ischemia of the heart followed by a 3 h reperfusion. Chronic pretreatment with oleuropein reduced the infarct size compared with the control (Andreadou et al., 2006). In doxorubicin-induced cardiomyopathy, oleuropein effectively improved the impaired cardiac geometry and function evidenced by transthoracic echocardiography (Andreadou et al., 2014). In isolated rat hearts, pretreatment with 20 μg/g oleuropein before ischemia resulted in a significant decrease in creatine kinase and reduced glutathione release in the perfusate. Reflow in ischemic hearts induced oxidized glutathione release and membrane lipid peroxidation, which were prevented by oleuropein. The reported data demonstrated the direct cardioprotective effect of oleuropein in the acute events that follow coronary occlusion, and the nutritional benefit of olive oil in the prevention of coronary heart disease (Weikl et al., 1996).

##### Panax notoginseng (Araliaceae)

The root of *Panax notoginseng*, known as Sanqi, Sanchi or Tianqi in East Asian countries, has been identified over 80 variants according to different substitute patterns. There are five main saponins R1, Rb1, Rg1, Rd, and Re, constituting up to 90% of the total *Panax notoginseng* used in the pharmacological experiment. A myriad of studies demonstrate the protective effect of notoginseng in cardiac injury. Firstly, it could significantly improve rats’ cardiac function evidenced by left ventricular ejection fractions, left ventricular fractional shortening, left ventricular dimensions at end diastole and left ventricular dimensions at end systole (Chen et al., 2011). Secondly, it could reduce infarct size and serum level of creatine kinase in rat models of myocardial ischemia (Yue et al., 2012; Han et al., 2013). Thirdly, it decreased serum levels of lactate dehydrogenase, cardiac troponin I, malondialdehyde and several cytokines, including TNF-a, IL-1β and C-reactive protein (Han et al., 2013). These findings suggest considerable therapeutic potentials of notoginseng for myocardial infarction. Interestingly, Notoginseng combined with the powdered extract of *Carthamus tinctorius*, an anti-thrombus herbal medicine, or with Sals, the active ingredient of *C. tinctorius*, showed stronger effects to reduce infarct size than either drug alone (Yue et al., 2012; Han et al., 2013; Liu et al., 2014). Thus, combination medication remedy should be considered for CVDs treatment.

##### Salviae miltiorrhizae (Lamiaceae)

In addition to hypertension and hyperlipidemia, the dried root of *Salviae miltiorrhizae* (Danshen) is widely used in China for the treatment of angina pectoris and acute ischemic stroke. It has a range of potentially beneficial effects, including improving microcirculation, causing coronary vasodilatation, suppressing the formation of thromboxane, inhibiting platelet adhesion and aggregation, and protecting against myocardial ischemia. Danshen is widely used either alone or in combination with other herbal ingredients for patients with coronary artery disease or CVDs. An acute myocardial infarction model was induced and both infarct size and echocardiographic response were evaluated at different time after surgery. Both Sal and tanshinone (two hypdrophilic and lipophilic compounds) delayed the development of ischemia by decreasing infarct size and improving systolic function post myocardial infarction (Wang et al., 2011). However, a recent review of randomized controlled trials of Danshen in ischemic disease published in mainland China identified 150 trials from 1998 to 2007, but concluded that the quality of these trials has not improved significantly over recent years and the overall quality is still poor (Yu et al., 2014). Since clinical studies have various methodological problems, further high-quality randomized controlled trials should be performed to assess the efficacy of this herb.

## Mechanisms of Plant Products in the Attenuation of CVDs

For over 2000 years, CVDs have been treated with numerous herbal remedies. However, these herbal remedies have not been well studied using modern cellular and molecular techniques. Based on the existing investigations into mechanisms of single herbal compounds, a summary of these studies is presented in the following section wherein the compounds are discussed according to their target site of activity.

### Vascular Smooth Muscle Cells

#### Inhibiting Expression or Activity of Contractile and Structural Proteins

The blood vessel tonicity is principally controlled by the contraction and relaxation of VSMCs. Upon stimulation, smooth muscle cells develop a contractile force by using the cross-bridge cycling between the contractile proteins actins and myosin initiated by the Ca^2+^-calmodulin interaction and modulated by many other proteins. Herbal medicine that regulates proteins or molecules in this pathway would modulate vascular contraction or relaxation. Firstly, blocking contractile and structural proteins may be one of the ways which natural plants could facilitate vasodilation and vascular remodeling. It has been shown that Tianma (the tuber of an orchid) enhances acetylcholine-induced vasorelaxation or phenylephrine-induced contraction in aortic rings (Feng et al., 2012). In more details, long-term treatment with small doses of Tianma resulted in a significant decrease of the expression levels of primary contractile protein actin to about half the level of the controls, as well as other cytoplasmic structural/cytoskeletal proteins, like Des, Vcl, Pdlim1 and Map4. Secondly, phosphorylation of myosin light chain protein is another target for herbs to determine vascular smooth muscle contraction. A10 cells, rat smooth muscle cells, were treated with kinds of herbs and it was observed that four herbs, Liu-Wei-Di-Huang-Wan, Jia-Wei-Xiao-Yao-San, Danshen and Gegen, reduced phosphorylation of myosin light chain, suggesting that these most common herbs may be beneficial for smooth muscle cell contractility (Lin et al., 2015).

#### Regulating Expression of Extracellular Matrix Proteins

Extracellular matrix is another target involved in vascular tone regulation. ECM proteins such as Postn is specifically induced upon tissue injury and could promote cellular adhesion and movement as well as collagen fibrillogenesis. ECM glycoproteins include Eln, Fbln5 and Prelp, being essential to maintain arterial morphogenesis and vessel elasticity. Tianma could induce up-regulation of ECM glycoproteins and down-regulation of ECM proteins in vascular system, thereby regulating blood vessel tonicity by increasing the arterial elasticity and stabilizing the arterial structure (Feng et al., 2012).

#### Regulating Calcium Levels in PKA/PKG/PKC-Dependent Way

Calcium levels in VSMCs also contribute to regulating the vascular contraction. Tetramethylpyrazine (TMP, also known as Ligustrazine), is a vasoactive component derived from Ligustium Wollichii Franchat. Using dog mesenteric arterial ring preparations, TMP not only caused a dose-dependent inhibition of vascular contractile responses to KCl and phenylephrine in solution with calcium, but also inhibited the responses to phenylephrine in Ca-free medium containing 50 μM EGTA. These results suggest that herbs may regulate vascular contraction or relaxation by the inhibition of Ca^2+^ influx, as well as the release of intracellular calcium (Kwan et al., 1990). Consistent with it, baicalein reduced AngII-, vasopression- and endothelin-stimulated increase in [Ca^2+^]_i_ of VSMCs (Saito et al., 1992). This effect was mimicked by a structurally unrelated lipoxygenase inhibitor, 5,8,11-eicosatriynoic acid and was restored by addition of 12(*S*)-hydroxyeicosatetraenoic acids (Saito et al., 1992). Thus, baicalein may be representative of the hypotensive herbs mediating Ca^2+^ levels which may be partly attributed to its inhibition of lipoxygenase and consequent decrease in biosynthesis and release of arachidonic acid-derived vasoconstrictor products.

Alterations in large-conductance Ca^2+^-activated K^+^ channel (BK_Ca_ channel) activity play a central role in mediating vasoconstriction and vasodilation. Baicalin (10–100 μM) not only concentration-dependently attenuated KCl-contracted mesenteric arteries, but also abolished selective BK_Ca_-channel inhibitor IbTX, voltage-dependent Ca^2+^ channel activator BayK8644, and PKC activator PMA-induced contractions at concentrations of 100 μM (Lin et al., 2010). These findings suggested that baicalin relaxes mesenteric artery via modulation of the BK_Ca_ and Ca^2+^ channels which may be mediated by the PKC associated pathway. Then, the BK_Ca_-mediated vascular contraction is further explored. The cAMP and the cGMP pathways are major regulators of smooth muscle contractility. By cyclic nucleotide assays, baicalin enhanced both cAMP and cGMP levels in mesenteric arteries. Based on the theory that smooth muscle relaxants that increase cAMP and cGMP have been shown to activate BK_Ca_ channels through direct phosphorylation of the channel proteins and through elevation of Ca^2+^ spark frequency, Lin et al. (2010) measured the effects of baicalin on BK_Ca_ current by whole-cell patch clamp electrophysiology. Baicalin was observed to enhance BK_Ca_ currents in a concentration-dependent manner, which were abolished by combining inhibitors of AC(SQ 22536) and sGC(ODQ), by combining inhibitors of PKA(KT5720) and PKG(KT5823), and PKC activator (PMA) in mesenteric artery smooth muscle cells. These results indicate that baicalin-induced mesenteric artery relaxations could be due to BK_Ca_ channel activation occurs not only as a result of PKA and PKG, but also as a result of cross-interaction with PKC (Lin et al., 2010).

#### Attenuating Proliferation and Migration of VSMCs

It’s well known that stimulation of VSMCs with TNF-α or platelet-derived growth factor-BB induced proliferation. *Camellia japonica* at concentrations 50, 100, 200, and 400 μg/mL significantly reduced the proliferation rate to 77.85, 70.12, 61.93, and 56.33% of the control (TNF-α treated without *Camellia japonica*) (Park et al., 2015). Similarly, Cinnamon extract inhibited the platelet-derived growth factor-BB-induced proliferation of VSMCs through a G0/G1 arrest, which down-regulated the expression of cell cycle positive regulatory proteins by up-regulating p21 and p27 expression (Kwon et al., 2015). The effects of Camellia japonica on VSMCs migration was evaluated with a wound healing assay. *Camellia japonica* concentration-dependently suppressed platelet-derived growth factor-BB induced VSMCs wound healing for 24 and 48 h after injury, indicating the migration inhibiting effects (Park et al., 2015). In addition, baicalein and baicalin induced anti-proliferative and anti-mitogenic effects in VSMCs of rabbit, rat and bovine were well summarized (Huang et al., 2005). These results provide additional evidence for the beneficial cardiovascular effects of natural products.

#### Anti-inflammation

Andrographolide is the most active and critical constituent isolated from the leaves of *Andrographis paniculata*, a herbal medicine widely used as an anti-inflammatory drugs in Asia. The mechanisms of the inflammatory effects of andrographolide in VSMCs were investigated by exposing VSMCs to a proinflammatory stimulus, TNF-α. Treating TNF-α-stimulated VSMCs with andrographolide suppressed the expression of inducible nitric oxide synthase (NOS), JNK, Akt, and p65 phosphorylation. However, it showed no effect on either IκBα degradation or p38 mitogen-activated protein kinase or ERK1/2 phosphorylation under these conditions. Both treatment with LY294002, a phosphatidylinositol 3-kinase/Akt inhibitor, and treatment with SP600125, a JNK inhibitor, markedly reversed the andrographolide-mediated inhibition of p65 phosphorylation. Thus, andrographolide-mediated inhibition of NF-κB activity in TNF-α-stimulated VSMCs occurs through the JNK-Akt-p65 signaling cascade mechanism, which is independent of IκBα degradation (Chen et al., 2014). These results collectively suggest that therapeutic interventions using andrographolide can benefit the treatment of vascular inflammatory diseases.

#### Improving Mitochondrial Function

In addition to cellular powerhouses, mitochondria are known as critical regulators of cell death. And they are the major cellular source of ROS which causes damage to mitochondrial DNA in human VSMCs in a number of cardiovascular pathologies. Thus, mitochondrial dysfunction may lead to the impairment of various aspects of tissue functioning. It has been known that cultured rat aortic VSMCs treated with AngII for 24 h exhibited mitochondrial dysfunction, including a decrease in mitochondrial oxygen consumption rates, ATP production and mtDNA levels, as well as the disruption of mitochondrial structural integrity (Lu et al., 2015). Together with the mitochondrial morphological changes, these alterations were reversed by Astragaloside IV, the major active ingredient of *Astragalus membranaceus*(Fisch.) Bge. (a traditional CHM). Moreover, treatment with Astragaloside IV also reversed the AngII-induced increase in the production of ROS, the increase in NADPH oxidase and xanthine oxidase activity, as well as the decrease in mitochondrial membrane potential and manganese-SOD activity. Furthermore, treatment with Astragaloside IV led to an increase in the mRNA and/or protein levels of peroxisome proliferator-activated receptor-gamma coactivator-1α (PGC-1α), mitochondrial transcription factor A, parkin and dynamin1-like protein1 in the VSMCs. These results indicate that Astragaloside IV exerts beneficial effects on AngII-induced mitochondrial dysfunction in rat VSMCs and that these effects are mediated through the inhibition of ROS overproduction, as well as the promotion of mitochondrial autophagy and mitochondrial biogenesis (Lu et al., 2015).

**FIGURE 1:**
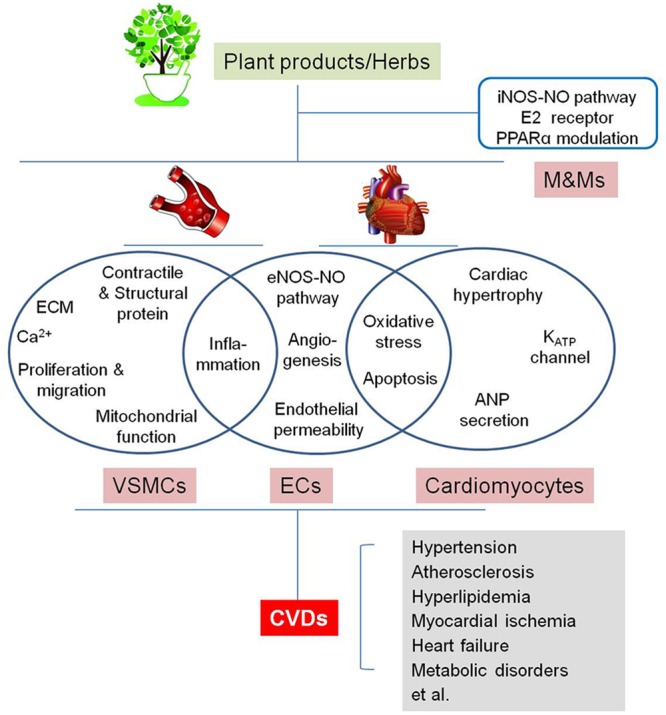
**A schematic diagraph indicating the targets on which plant products/herbs act during the pathogenesis of CVDs.** Plant products/herbs demonstrate protective effect in CVDs by attenuating damage in cardiomyocytes, endothelial cells (ECs), vascular smooth muscle cells (VSMCs) and macrophages/monocytes (M&Ms). In VSMCs, plant products/herbs show the beneficial effect by inhibiting expression or activity of contractile and structural proteins, modulating expression of ECM proteins/glycoproteins, regulating calcium levels, attenuating proliferation and migrations, alleviating inflammation, and improving mitochondrial function. In ECs, plant products/herbs show the beneficial effect by inhibition of inflammation, oxidative stress and apoptosis, activation of endothelial nitric oxide synthase(NOS)-NO signaling pathway, induction of angiogenesis and suppression of endothelial permeability. In cardiomyocytes, plant products/herbs show the beneficial effect by inhibiting cardiac hypertrophy, oxidative stress and apoptosis, opening K_ATP_ channels and increasing atrial natriuretic peptide (ANP) secretion. In macrophages and monocytes, plant products/herbs show the beneficial effect by inhibition of inducible NOS-NO signaling pathway, activation of estrogen receptor and nuclear receptor peroxisome proliferator activated receptor alpha (PPARα). A myriad of molecular, and cellular pathways are favorably modulated by plant products/plants or their extracts.

Overall, herbal medicines discussed here do appear to show pharmacological effects *in vitro* and in animal studies, which may influence CVDs. In VSMCs, these natural plants exert the protective effect by a series of processes (**Figure 1**) which include inhibiting expression or activity of contractile and structural proteins, modulating expression of ECM proteins/glycoproteins, regulating calcium levels, attenuating proliferation and migrations, alleviating inflammation, and improving mitochondrial function.

### Endothelial Cells

#### Activation of NO Signaling Pathway

Endothelial cells, which constitute the inner cellular lining of blood vessels, have a key role in regulating vascular homeostasis and function, such as vasorelaxation, vascular integrity, and local inflammation. Endothelium-dependent vasodilation was commonly applied to examine the endothelium function, in which NO is a potent vasodilator and plays an important role in regulating vascular tones. Some herbal medicine functions by targeting at the NO producing process. In studies at the arterial levels, both *Lysimachia clethroides* and gypenosides elicit vasorelaxation which was abolished by endothelial NOS (eNOS) or guanylyl cyclase inhibitors (Tanner et al., 1999; Lee et al., 2010). Sal B, a hydrophilic caffeic acid derivative of Danshen, exerts an important regulatory function on endothelial tissue of isolated mouse aorta by promoting NO production through the inhibition of arginase activity (Joe et al., 2012). Fo Shou San, an ancient herbal decoction (Chuanxiong Rhizoma and Angelicae Sinensis Radix Danggui in a ratio of 2:3), reversed homocysteine-induced impairment of acetylcholine-evoked endothelium-dependent relaxation in rat aortic rings (Bi et al., 2012). In studies at the cellular levels, gypenosides induced a concentration-dependent increase in NO production from cultured bovine endothelial cells (Tanner et al., 1999). Consistent with it, magnesium tanshinoate B (another compound purified from Danshen) or Fo Shou San, also stimulated the release of NO and its metabolites from a human endothelial cell line (ECV304) (O et al., 2000; Bi et al., 2012). By exploring the mechanisms by which NO production is induced, it was observed that the cellular NOS activities were significantly enhanced with a concomitant increase in the levels of constitutive NOS protein mass (O et al., 2000), phosphorylation levels of serine^1177^-eNOS and serine^473^-Akt (Lee et al., 2010; Lu et al., 2010; Bi et al., 2012) by a series of herbal products treatment, including magnesium tanshinoate B, lysimachia clethroides, nordihydroguaiaretic acid (main metabolites of creosote bush) and Fo Shou San. In addition, Fo Shou San elevated intracellular Ca^2+^ levels and eNOS phosphorylation, which was inhibited by the Ca^2+^ chelator BAPTA-AM (Bi et al., 2012). These results indicate herbals may benefit endothelial function through increased activity of Akt kinase and eNOS, which is via a rise of intracellular Ca^2+^.

#### Inhibition of Inflammation

Inflammation plays a crucial role in the pathophysiology of CVDs, in which adhesion molecules, pro-inflammatory cytokines and chemokines are involved. The anti-inflammatory effects of Danshen and its active ingredients (Sal B, Tanshionone IIA, Protoc et al.) were extensively investigated in traditional Chinese medicine for therapy of CVDs. Incubation with TNFα or ADP is widely used to induce inflammation *in vitro*. In TNFα treated human coronary artery endothelial cells, Sal B significantly inhibited the inflammation and decreased matrix metalloproteinase-9 expression and activity by blocking the activation of IκBα/NFκB through JNK and ERK1/2 signaling pathways (Ma et al., 2015). In TNFα treated HUVECs, although neither Sal B nor Tanshinone IIA inhibit the inflammation (Stumpf et al., 2013), Danshen and Protoc attenuated expression and/or release of CD40, VCAM-1 and ICAM-1 as well as cytokines or chemokines (IL-6, IL-8 and MCP-1) (Stumpf et al., 2013). In ADP-treated human platelets, pre-treatment with both Sal B, Tanshionone IIA and Danshen significantly attenuated platelet P-selectin expression (Stumpf et al., 2013). Gypenoside XLIX, a dammarane-type glycoside, is a prominent component of *G. pentaphyllum*. Studies demonstrate that Gypenoside XLIX inhibits TNFα-induced VCAM-1 over expression and hyperactivity in human endothelial cells via a PPARα-dependent pathway (Huang et al., 2007a). These findings provide useful insight into the rational basis of CHM in the treatment of CVDs.

#### Attenuation of Oxidative Stress

It’s well known that oxidative stress contributes to endothelium dysfunction. There are a myriad of herbs that demonstrate effects of endothelial cell protection by different mechanisms. For example, Sal B decreases ROS production in the aortic rings. Since ROS easily acts with NO to produce peroxynitrite anions, which is a potent and potentially toxic oxidant that damages various types of biomolecules, it can be deduced that Sal B may exert its protective function by precluding NO consumption through adverse reactions such as peroxynitrite formation and preserving NO bioavailability (Joe et al., 2012). EGb 761 (Ginkgo biloba extract) suppresses oxidative stress in a dose-dependent manner in high glucose-stimulated HUVEC. In addition to inhibition of ROS generation and 8-OHdG content, it attenuated oxidative DNA damage, indicating herbs could exert endothelial protection by alleviating endothelial DNA oxidation (He Y.T. et al., 2014). *Echium amoenum* extract (a major source of anthocyanins) demonstrated antioxidant and cytoprotective effect in H_2_O_2_-treated HUVECs. In the endothelial injury model, pretreatment of HUVECs with the anthocyanin-rich extract at concentrations of 100–1000 μg/ml reduced the cell death, decreased hydro peroxide concentration, and increased ferric reducing antioxidant power value in both intra- and extra-cellular fluid in a concentration-dependent manner (Safaeian et al., 2015). KRGE reduces the H_2_O_2_-induced cell injury in HUVECs too. The inhibited effect on cell death was blunted by HO-1 inhibitor zinc protoporphyrin. HO-1 is considered to augment the cellular defense against various agents inducing cytotoxic injury. KRGE was observed to induce up-regulation of HO-1 expression in HUVECs, which was abolished by specific silencing of Nuclear factor-eythroid 2-related factor 2 (Nrf2, a important anti-oxidant transcription factor) expression (Yang et al., 2011). These results suggest that Korean Red Ginseng may exert a vascular protective effect through Nrf2-mediated HO-1 induction in human endothelial cells. Ophiopogonin D is one of the most bioactive components of Radix Ophiopogon japonicas. Its pretreatment showed a series of antioxidation-related protective effects: (1) reducing H_2_O_2_-induced lipid peroxidation and protein carbonylation, (2) attenuating mitochondrial ROS generation and cell apoptosis, (3) restoring cellular total antiboidative capacity, (4) inhibiting the release of inflammatory cytokines and blocking activation of NFκB and ERK signaling cascades, (5) suppressing the enzymatic activity of catalase, HO-1, and caspases (Qian et al., 2010). In general, these findings support the protective role of herbs as an effective antioxidant in endothelial injury.

#### Inhibition of Apoptosis

Although endothelial cell dysfunction occurs in many different disease processes, caspase-dependent apoptotic cell death induced by the extrinsic or intrinsic pathways is identified as a common denominator. Protective effect of KRGE was examined in a serum-deprived apoptosis model, which demonstrated that it could prevent serum deprivation-induced HUVEC apoptosis (Kim et al., 2013). This effect was mediated by increased Bcl-2 and Bcl-XL protein expression, PI3K/Akt-dependent Bad phosphorylation, and eNOS/NO-mediated *S*-nitrosylation of caspases. Cytosolic cytochrome *c* is known to play a crucial role in the activation of the initiator caspase-9 and the effector caspase-3. KRGE prevented subcellular redistribution of cytochrome c from the mitochondrion into the cytosol, resulting in suppression of the extrinsic pathway of apoptosis activation (Kim et al., 2013). The cytoprotective property of KRGE may be valuable for developing new pharmaceutical means that limit endothelial cell death induced during the pathogenesis of vascular diseases.

#### Inducing Angiogenesis

Angiogenesis, the formation of new blood vessels from preexisting endothelium, is critical to a variety of processes, both physiologically (embryonic development and wound healing) and pathologically (malignancy and chronic inflammation). It is a complex biologic function comprising several steps, including sequential basement membrane degradation, endothelial cell migration and proliferation, tube formation, inhibition of endothelial proliferation, and the stabilization of new vessels. Modulation of any of these steps would affect new vessel formation. Scutellarin, a known flavone glycoside, is the primary active component of the traditional CHM Erigeron breviscapus (Vant.) Hand. Mazz. It has been shown to induce endothelial cell proliferation and migration, promote capillary-like tube formation, and significantly upregulate platelet endothelial cell adhesion molecule-1 (Gao Z.X. et al., 2010). These results suggest that herbal medicine promotes angiogenesis and may form a basis for angiogenic therapy.

#### Suppression of Endothelial Permeability

Endothelial cell hyperpermeability is another factor implicated in CVDs, in which the importance of tyrosine phosphorylaton and kinase activity in oxidant-mediated loss of cell junction structures has been demonstrated. Extract of salvia miltiorrhiza and its major ingredients, Danshensu and Sal B inhibited TNFα-induced endothelial permeability, evidenced by attenuated junctional protein phosphotyrosine and prevention of beta-catenin disassociation from the cytoskeleton (Ding and Yuan, 2007). The mechanism of pharmacological action was further explored. The extract, Danshensu and Sal B also repressed expression of vascular endothelial growth factor and ERK activation in TNFα treated HUVEC cells. In addition, it was found that the extract attenuated the disorganization of vascular endothelial-cadherin, which is likely due to a reduction of vascular endothelial growth factor protein expression as a result of modulation of the ERK signaling pathway (Ding et al., 2005). These findings suggest that active herbal ingredients may help to attenuate CVDs by maintaining the integrity of endothelial junction structure.

In general, herbal medicines do show the beneficial effects in endothelial cells. As shown in **Figure 1**, these natural plants exert the protective effect by inhibition of inflammation, oxidative stress and apoptosis, activation of eNOS-NO signaling pathway, induction of angiogenesis and suppression of endothelial permeability.

### Cardiomyocytes

#### Alleviation of Cardiac Hypertrophy

Pathological cardiac hypertrophy induced by increased sympathetic drive can subsequently lead to congestive heart failure, which represents the major cause of morbidity and mortality worldwide. *Astragalus* polysaccharide is an active compound extracted from Chinese herb *Astragalus membranaceus* for “Qi-invigorating.” In the *in vitro* cardiac hypertrophic model induced by isoprenaline, *Astragalus* polysaccharide treatment inhibited significant increases in cell surface area, total protein content, protein synthesis as well as the expression of hypertrophic markers, including ANP and B-type natriuretic peptide. In addition, *Astragalus* polysaccharide pretreatment not only alleviated the augmentation of intracellular free calcium during cardiac hypertrophy but also upregulated expression of calcineurin, translocation of nuclear factor of activated T cells, cytoplasmic 3 into nucleus and activation of calmodulin kinase II (reflected by p-CaMKII) (Dai et al., 2014). According to this research, *Astragalus* polysaccharide exerted its anti-hypertrophic action via inhibiting Ca^2+^-mediated calcineurin/NFATc3 and CaMKII signaling cascades, which provided new insights into the application of *Astragalus* polysaccharide to the therapy of heart diseases.

Chlorogenic acid is an important component of CHM, which acts as an antioxidant scavenging free radicals and preventing inflammation. Pre-treatment of chlorogenic acid in the isoprenaline-induced neonatal rat myocytes, decreased the levels of the hypertrophic markers, ANP, B-type natriuretic peptide and β-MHC. The level of the intracellular ROS was reduced and the nuclear translocation of NF-κB was blocked, whereas NF-κBIA, an inhibitor of NF-κB, was upregulated accordingly. These data reveal that chlorogenic acid may inhibit cardiac hypertrophy by attenuating NF-κB signaling pathway and suppressing ROS production (Li et al., 2014).

#### Attenuation of Oxidative Stress

Many data have demonstrated that ROS production by myocardial endothelial mitochondria contributes to heart disease and oxidative stress within ventricular myocytes can also be detrimental to the heart. In fact, much of the contractile dysfunction and adverse myocardial remodeling, which has been observed in a wide range of cardiomyopathies, involves oxidative stress and eNOS uncoupling leading to a decrease in NO. There are a series of targets at which herbal medicine act to improve myocardial endothelial function by attenuation of oxidative stress.

(1) Intracellular generation of ROS, including superoxide, hydrogen peroxide and hydroxyl radicals, contributes to the pathogenesis of cellular injury during ischemia and reperfusion in cardiac tissues. Thus, free radical scavenging and inhibition of oxidases will exert protective effect in cardiac injury. Baicalein may provide such therapy by deletion of free radicals, reducing hydroxyl radicals generation by suppressing iron-catalyzed Fenton reaction or decreasing ROS production by inhibiting activities of NADH-oxidase and xanthine oxidase (Huang et al., 2005). (2) ROS may reduce NO bioactivity through the formation of peroxynitrite and NOx. Thus it is easy to understand the oxidized LDLs-induced endothelial dysfunction could be restored by L-arginine and NO. The olive products constitute a rich source of polyphenols such as oleuropein and its derivatives, including hydroxytyrosol, which scavenge free radicals and inhibit the chemical oxidation of LDL. Studies have shown that treatment with high doses of oleuropein at min 1 of reperfusion significantly reduced levels in plasma NOx in hypercholesterolemic rabbits coupled with reduction of infarct size in this group (Andreadou et al., 2014). Therefore, plant products may function as a NO donor to inhibit formation of peroxynitrite and NOx. (3) Circulating or cellular levels of malonaldehyde, TBARS, and protein content in carbonyls serve as Lipid peroxidation and general oxidative stress. The decrease in these biomarkers indicates the protective action of oleuropein, baicalein, crocetin, *Allium chinense* and *Astragalus* against oxidative stress by scavenging free radicals (Nasa et al., 1993; Huang et al., 2005; Shen and Qian, 2006; Ren et al., 2010; Andreadou et al., 2014). (4) A great amount of ROS generates from mitochondrial electron transport inhibition, which induces lethal oxidant damage of cardiomyocytes. Qian-Kun-Nin, a CHM formulation was observed to significantly decreased cell death and attenuate oxidation of DCFH in cells exposed to the mitochondrial site III inhibitor, antimycin A, consistent with a decrease in oxidative stress (Shao et al., 2001). These findings indicate herbal medicine may inhibit oxidative stress by improving mitochondrial function. (5) Myocardial SOD, glutathione peroxidase and myocardial catalase are the main antioxidant enzymes, which are dramatically reduced after ischemia and reperfusion. Pretreatment with oleuropein, *Astragalus*, crocetin or cynaroside kept the SOD activity stable in myocardial infarction models or norepinephrine induced cardiac hypertrophy and therefore left the myocardium in an “antioxidant state”(Nasa et al., 1993; Shen and Qian, 2006; Sun et al., 2011; Andreadou et al., 2014). Thus, keeping the function of antioxidant enzymes standards for another target to inhibit oxidative stress and protect cardiac injury. (6) Nrf2 is a master transcription factor of endogenous anti-oxidative defense systems. Herbs such as American ginseng could enhance this antioxidant system in rat cardiac H9C2 cells (Li et al., 2010). The antioxidant systems include Nrf2 protein expression, Nrf2 nuclear translocation, Nrf2 transcriptional activity, direct Nrf2 binding to its target gene promoters, and expression of a group of anti-oxidative genes. Thus, herbs may serve as antioxidant enhancer to provide cardioprotection against pathological cardiac injury and remodeling. (7) Doxorubicin triggered impaired left ventricular contractility and inflammatory and degenerative pathology lesions, indicating a successful cardiomyopathy model. Metabonomic analysis revealed treatment of oleuropein induced an favorable variations in the ratio of glycolytic end products (alamine + lactate) to end products of lipid metabolism (acetate), suggesting restoration of the impairment in aerobic glucose metabolism and cardioprotection of ischemia (Andreadou et al., 2014). This may help indentifying novel therapeutic targets in the effect of plant products to protect the heart from injury.

#### Inhibition of Apoptosis

H_2_O_2_ treatment induces both oxidative damage and apoptosis, contributing to cardiac injury in cultured rat cardiomyocytes. Cynaroside, a flavonoid compound, has been shown to enhance the endogenous anti-oxidative activity, thereby inhibiting intracellular ROS generation. It also showed the protective effects against H_2_O_2_-induced apoptosis in H9c2 rat cardiomyoblasts. The mechanism by which cynaroside reduced the apoptotic rate were further explored. Cynaroside pretreatment not only moderated H_2_O_2_-induced disruption of mitochondrial membrane potential, but also increased the expression of anti-apoptotic protein Bcl-2 while decreased the expression of pro-apoptotic protein Bax, and thereby inhibited the release of apoptogenic factors (cytochrome c and smac/Diablo) from mitochondria in H9c2 cells. Moreover, cynaroside pretreatment showed an inhibitory effect on the H_2_O_2_-induced increase in JNK and P53 protein expression (Sun et al., 2011). These findings suggest that flavonoid products prevent cardiomyocytes apoptosis *in vitro* by reducing the endogenous production of ROS, maintaining mitochondrial function, and modulating the JNK and P53 pathways.

#### Opening K_ATP_ Channels

Cardiac myocyte is the cell types in which K_ATP_ channels were originally discovered. It is well established that K_ATP_ channels are present at high density in the sarcolemma of cardiac myocytes where they link membrane excitability with the cellular bioenergetic state. The opening of these channels in the heart is believed to mediate ischemic preconditioning, a phenomenon whereby brief periods of ischemia/reperfusion protect the heart against myocardial infarction. Pinacidil is a potassium channel opener which mediate preconditioning at the beginning of sustained hypoxia by opening of sarcolemmal K_ATP_ channels (Budas et al., 2004). Similar to it, Guanxinkang treatment benefit the heart function by increasing mRNA and protein expression of K_ATP_ subunits (Kir6.1, Kir6.2, SUR2A, SUR2B) in ventricular myocytes (Chen et al., 2010). Keep in line with it, extracts of *Astragalus* demonstrated its anti-apoptotic effect in H_2_O_2_-injured cardiomyocytes only when the K_ATP_ channels were open, whereas the protective effect was not observed when the channels were inhibited by the K_ATP_ channel blocker (5-HD, glibenclamide) (Nasa et al., 1993). These findings suggest enhancing the open of K_ATP_ channel is one of the important mechanisms by which herbs protect myocardial cells from ischemic injury.

#### ANP Secretion

Atrial natriuretic peptide is secreted by the heart atrium cells. ANP binds to a specific set of receptors and acts to reduce the water, sodium and adipose loads on the circulatory system, thereby reducing cardiac output and systemic BP. Emodin, an active anthraquinone constituent isolated from the rhubarb, was observed to increase ANP secretion concomitantly with a decrease in atrial pulse pressure and stroke volume in a concentration-dependent manner. Inhibition of K^+^ channels with tetraethylammonium and glibenclamide or inhibition of L-type Ca^2+^ channels with nifedipine, attenuated the emodin-induced changes in ANP secretion and atrial dynamics (Zhou et al., 2014). These findings demonstrate that emodin may increase ANP secretion via inhibition of L-type Ca^2+^ channels through an activation of K_ATP_ channel in isolated beating rabbit atria. This study provides a rationale for the use of herbal medicine in the treatment of impairment of the regulation of the cardiovascular homeostasis.

To summarize, plant products/herbal medicines do protective cardiomyocytes from injury. As shown in **Figure 1**, plant products/herbs show the beneficial effect by inhibiting cardiac hypertrophy, oxidative stress and apoptosis, opening K_ATP_ channels and increasing ANP secretion.

### Macrophages and Monocytes

#### Inhibition of iNOS-Mediated NO Production

In the milieu of cardiovascular risk factors that disturb vascular homeostasis, inflammation represents a key early event in vascular pathology, in which monocytes activation, adhesion to the endothelium and infiltration of macrophages into blood vessels are thought to play important pathogenic roles in atherosclerosis and other inflammatory CVDs. It’s well known immune cells-derived excessive production of NO by inducible NOS (iNOS) can cause endothelial damage, leading to multiple vascular wall injuries. The transcriptional and translational regulation of iNOS in various cell types can be induced by cytokines, growth factors, and endotoxins. Lipopolysaccharide (LPS) stimulation has been shown to enhance iNOS expression, NO production, and macrophage arginase activity in RAW 264.7 macrophages, which were inhibited by Danshen extracts and Sal B. In addition, cytoprotective molecule HO-1 expression was upregulated by these Danshen products too (Joe et al., 2012). SnPP is well characterized as a potent competitive inhibitor of HO activity. Since the iNOS expression, NO production, and TNFα production, were completely abolished by SnPP, the anti-inflammatory effect of Sal B in macrophages may be related to modulation of HO-1. Hb serves as a CO scavenging compound. Preincubation of Hb for 30 min reversed the inhibitory effect of Sal B on LPS-induced iNOS expression, NO production and NFκB activation in the RAW 264.7, indicating the regulatory effect of Sal B on inflammation may be through mediating the formation of CO (Joe et al., 2012), which shares the similar mechanism to HO-1 down-regulating proinflammatory cytokines production in LPS-stimulated macrophages (Otterbein et al., 2000). Although the mechanism by which HO-1 down-regulates iNOS is incompletely clear, it may involve transcriptional inhibition through CO formation or reduction of heme bioavailability for iNOS synthesis.

#### Activation of Estrogen Receptor

Consistent with the effect of Sal B, Tanshinone IIA (another major compound extracted from Danshen) exerts its anti-inflammatory effects by inhibition of iNOS gene expression and NO production, as well as inhibition of inflammatory cytokine (IL-1β, IL-6 and TNFα) expression in RAW 264.7 cells (Fan et al., 2009). Since Tan IIA has a similar structure to that of 17β-estradiol, the estrogenic activities was examined in the immune cells. The Tanshinone IIA’s anti-inflammatory effects was mimicked by estradiol and blocked by ICI182187, the antagonist of estrogen receptor (Fan et al., 2009). In fact, the most commonly used alternative herbal medicines for estrogen replacement are soy, black cohosh, dong quai and ginseng. Thus, these herbal medicines may serve as a potential selective estrogen receptor modulator to treat inflammation-associated CVDs without increasing the risk of breast cancer.

#### Activation of PPARα

Activation of the nuclear receptor, PPARα, has been demonstrated to modulate many aspects of lipoprotein metabolism and inflammation both *in vitro* as well as *in vivo*. The tissue distribution of PPARα is extensive and it may mediate many of anti-inflammatory and anti-atherogenic effects. Gypenoside XLIX, a dammarane-type glycoside, is one of the prominent components in *Gynostemma pentaphyllum*. It has been identified as a potent PPARα activator in HUVEC study (Huang et al., 2007a). Tissue factor is involved in many diseases including CVDs and hence may be an attractive target for directed CVDs therapeutics. In human monocytic THP-1 cells transfected with promoter reporter constructs pTF-LUC, Gyp XLIX (0–300 mM) concentration-dependently inhibited LPS-stimulated tissue factor promoter activity, mRNA and protein overexpression. This effect was similar to those of Wy-14643, a potent synthetic PPARα activator, and completely abolished in the presence of the PPARα selective antagonist MK-886 (Huang et al., 2007b). These data indicate that Gyp XLIX inhibits LPS-induced tissue factor overexpression and enhancement of its activity in human THP-1 monocytic cells via PPARα-dependent pathways, providing new insights into the mechanism of the Chinese herbal plants in CVDs treatment.

Overall, the natural plants/herbs do demonstrate inhibition of inflammation in macrophages and monocytes. These effects are achieved by inhibition of iNOS-NO signaling pathway, which may be through activation of estrogen receptor and nuclear receptor PPARα-dependent signaling pathways (**Figure 1**).

## Conclusion

Although many studies have been missed in this review due to our search strategy and the limited access to some articles, the aforementioned evidence is strongly indicative of the notion that herbs/natural plants are the emerging medicine in the prevention and/or treatment of CVDs. One may predict that herbal remedies will receive even more attention in the coming years. However, some objective limitations should be considered based on the existing literature. Firstly, the CHM are widely studied in China. Accordingly, many articles published on the Chinese literature which may limit these work to be retrieved and public to the world. In addition, a location bias cannot be excluded since trials published in local journals are more likely to report significant results than those published in worldwide mainstream medical journals. Secondly, many of the clinic trials were not well-designed and with poor methodological quality by lacking formal inclusion/exclusion criteria, inadequate description of the randomization procedures, too short duration of therapy and follow-up to achieve conclusive results, et al. Poorly designed and reported clinic trails usually exaggerate the treatment effects which will misled decision making in clinic. Thirdly, compounds contained in one herb, even in an extract of one herb are very complicated. Thus, it is a very tough work to clarify the mechanism of plant products/herb for treating CVDs and interaction with other medicines (including western medicines). Fourthly, many herbal medicine remedies used today have not undergone careful scientific assessment and there still lack preclinical study on the side effects, toxic effects and major drug-to-drug interactions in record. Even though, natural plants/herbs are germs of medicine and should deserve more attention and application. Therefore, to develop new agents with effectiveness and safety from traditional Chinese medicine is a promising way for prevention and treatment of patients with CVDs. However, clinic study criteria should be documented to standardize evaluation of plants/herbs. In addition, international collaboration may be encouraged, promoted and financed from the governments in order to improve the overall research quality.

## Author Contributions

CL drafted the work; YH revised the manuscript. Both CL and YH contributed substantially to the conception or design of the work, approved the final version to be published, and agreed to be accountable for all aspects of the work in ensuring that questions related to the accuracy or integrity of any part of the work are appropriately investigated and resolved.

## Conflict of Interest Statement

The authors declare that the research was conducted in the absence of any commercial or financial relationships that could be construed as a potential conflict of interest.

## Abbreviations

ANP: atrial natriuretic peptide
BP: blood pressure
BK_Ca_: channel Ca^2+^-activated K^+^ channel
CHM: Chinese herbal medicine
CVDs: cardiovascular diseases
ECM: extracellular matrix
(e)NOS: (endothelium) nitric oxide synthase
HDL-C: high-density lipoprotein cholesterol
HO: heme oxygenase
HUVEC: human umbilical vein endothelial cells
IL: interleukin
K_ATP_: ATP-sensitive potassium
KRGE: Korean Red Ginseng extract
LDL: low-density lipoprotein
LDL-C: low-density lipoprotein cholesterol
NO: nitric oxide
PPARα: peroxisome proliferator-activated receptor-alpha
ROS: reactive oxygen species
Sal: salvianolic acid
SHR: spontaneous hypertension rats
SOD: superoxide dismutase
TC: total cholesterol
TG: triglyceride
VSMCs: vascular smooth muscle cells
